# Calibration to maximize temporal radiometric repeatability of airborne hyperspectral imaging data

**DOI:** 10.3389/fpls.2023.1051410

**Published:** 2023-02-13

**Authors:** Christian Nansen, Hyoseok Lee, Anil Mantri

**Affiliations:** Department of Entomology and Nematology, University of California, Davis, Davis, CA, United States

**Keywords:** optical sensing, radiometric calibration, spectral repeatability, reflectance profiling, image-based classification

## Abstract

Many studies provide insight into calibration of airborne remote sensing data but very few specifically address the issue of temporal radiometric repeatability. In this study, we acquired airborne hyperspectral optical sensing data from experimental objects (white Teflon and colored panels) during 52 flight missions on three separate days. Data sets were subjected to four radiometric calibration methods: no radiometric calibration (radiance data), empirical line method calibration based on white calibration boards (ELM calibration), and two atmospheric radiative transfer model calibrations: 1) radiometric calibration with irradiance data acquired with a drone-mounted down-welling sensor (ARTM), and 2) modeled sun parameters and weather variables in combination with irradiance data from drone-mounted down-welling sensor (ARTM+). Spectral bands from 900-970 nm were found to be associated with disproportionally lower temporal radiometric repeatability than spectral bands from 416-900 nm. ELM calibration was found to be highly sensitive to time of flight missions (which is directly linked to sun parameters and weather conditions). Both ARTM calibrations outperformed ELM calibration, especially ARTM2+. Importantly, ARTM+ calibration markedly attenuated loss of radiometric repeatability in spectral bands beyond 900 nm and therefore improved possible contributions of these spectral bands to classification functions. We conclude that a minimum of 5% radiometric error (radiometric repeatability<95%), and probably considerably more error, should be expected when airborne remote sensing data are acquired at multiple time points across days. Consequently, objects being classified should be in classes that are at least 5% different in terms of average optical traits for classification functions to perform with high degree of accuracy and consistency. This study provides strong support for the claim that airborne remote sensing studies should include repeated data acquisitions from same objects at multiple time points. Such temporal replication is essential for classification functions to capture variation and stochastic noise caused by imaging equipment, and abiotic and environmental variables.

## Introduction

Optical sensing, machine vision, and remote sensing are common terms referring to the process of acquiring optical signals to detect and classify individual “objects” into pre-defined categories or along continuous gradients. Across spatial scales and types of optical sensors, classifications of remote sensing data are based on the fundamental assumption that unique and detectable “optical traits” within optical signals can be acquired consistently and used accurately to identify and/or characterize objects. As an example, a thorough and well-performed study concluded that reflectance values in spectral bands near 700 nm provide quase-universal indication of abiotic and biotic stress in plants (Carter and Knapp, 2001). Importantly, non-stressed plants also have reflectance near 700 nm, so the optical classification challenge is to identify and quantify what would be considered non-stress reflectance at 700 nm in order to use this spectral information as an optical trait to detect and diagnose plant stress. Simply stated and when objects are classified into categories, it is assumed that between-category difference of optical signals is greater than within-category variation.

**Figure 1:**
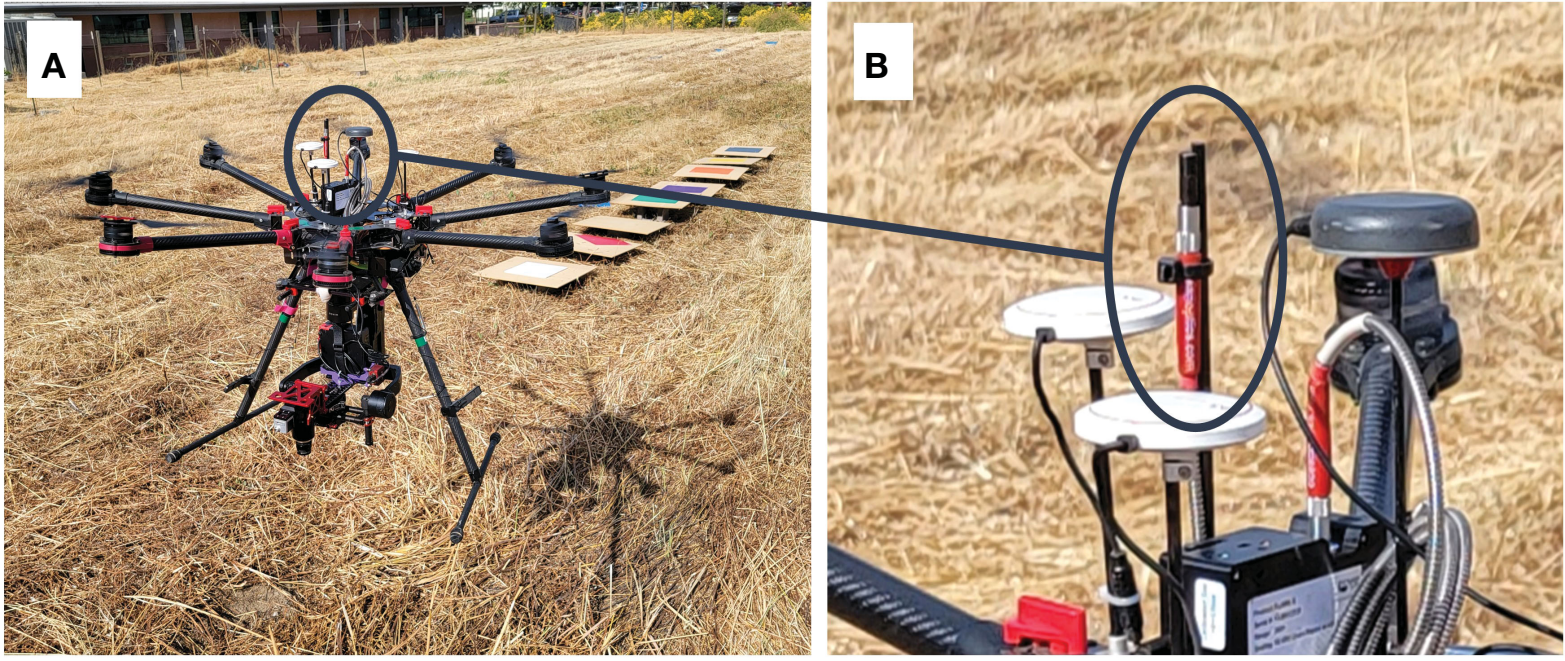
Octocopter drone system used in this study **(A)** with down-welling irradiance sensor **(B)**.

Within-category variation may be viewed as noise or error, and several sources of error adversely affect classification of objects based on airborne remote sensing data (Anderson and Peleg, 2007; Schott, 2007; Hruska et al., 2012; Zhang and Kovacs, 2012; Aasen et al., 2018). In airborne remote sensing studies and applications, sources of error are markedly influenced by spatio-temporal dynamics of sun parameters (i.e. altitude, azimuth, and distance from earth) and atmospheric conditions. Specific studies have measured and modeled influence of solar irradiance and angle as functions of time of day (King et al., 1997) and effects of atmospheric contributions and drone flight configurations on airborne remote sensing data sets (Kedzierski et al., 2019; Poncet et al., 2019; Zarzar et al., 2020). Radiometric calibration is performed to minimize optical signal noise induced by dynamics of sun parameters and atmospheric conditions, and it can be accomplished *via* deployment of stationary reference objects, which is referred to as vicarious calibration or empirical line method (ELM) (Smith and Milton, 1999; Karpouzli and Malthus, 2003; Baugh and Groeneveld, 2008; Del Pozo et al., 2014; Wang and Myint, 2015; Aasen et al., 2018; Iqbal et al., 2018; Mafanya et al., 2018; Poncet et al., 2019; Agapiou, 2020; Zarzar et al., 2020). Several articles have provided comprehensive discussions of ELM calibration and listed important underlying assumptions when this radiometric calibration method is used (Smith and Milton, 1999; Baugh and Groeneveld, 2008; Aasen et al., 2018). Alternatively, radiometric calibration may be based on solar and atmospheric modeling (atmospheric radiative transfer models, ARTMs) (Biggar et al., 1994; Aasen et al., 2018; Poncet et al., 2019). ARTM calibration is based on parameterization of incident irradiance and potentially other variables, including time of day, date, location, and weather conditions (Aasen et al., 2018), and the basic goal is to generate an estimate of Lambertian radiance, which is then used as reference. Moreover, radiance signals from target objects are divided with ARTM calibration estimates of Lambertian radiance to obtain reflectance. Thus, the fundamental difference between ELM and ARTM calibration is whether to use an actual calibration board, such as, white Teflon in ELM calibration or to use a virtual or model-based reference (ARTM for radiometric calibration. A key challenge in ARTM calibration is accurate measurement or theoretical calculation of incident irradiance (Smith and Milton, 1999), but possible solutions include deployment and integration of a stationary reference spectrometer or sun photometer (Zarco-Tejada et al., 2012; Burkart et al., 2013; Del Pozo et al., 2014), or irradiance data acquisition with a drone-mounted down-welling sensor (Nevalainen et al., 2017; Mamaghani and Salvaggio, 2019). As an example, **Figures 1A, B** show the optical drone system used in this study. Through software control and integration, down-welling irradiance data are acquired as a separate file (but concurrently with optical sensing data of objects) and later used as white balance to convert radiance data from target objects into reflectance.

Several articles have highlighted importance of low radiometric repeatability and its adverse effect on accuracies of optical sensing classification functions (Peleg et al., 2005; Baghzouz et al., 2006; Anderson and Peleg, 2007; Vilaseca et al., 2014; Nansen and Elliott, 2016). In a few studies, radiometric repeatability was examined experimentally manipulated to determine its relative effect on accuracy of classification functions (Nansen et al., 2019; Nansen et al., 2022). Effective and user-friendly radiometric calibration methods are needed to minimize within-class optical noise and to maximize between-class differences in airborne remote sensing data sets. However, it is surprisingly rare that studies of radiometric calibration methods include a temporal component as part of optimizing what is here referred to as “temporal radiometric repeatability”, *T_r_*, which we defined as:


$$Equation\;1:T_{r}=\;100-\;\left\{\frac{Confid_{95}\;\times 100}{Signal_{avg}}\right\}$$

In which, it is assumed that optical sensing data (i.e. radiance or reflectance) in individual spectral bands follow a normal distribution when an object is imaged multiple times. Moreover, we calculated 95% confidence intervals (*Confid_95_*) for each spectral band and divide with average signal value (*Signal_avg_*). This ratio is converted into percentage, and finally subtracted from 100. Accordingly, 100 equals the maximum radiometric repeatability, and due to standardization (division with average signal value), it can be compared across spectral bands with varying signal intensities. Peleg et al. (2005) boldly and eloquently stated the following: “*Hyperspectral image cubes acquired in consecutive flights over the same target should ideally be identical. In practice, two consecutive flights over the same target usually yield significant differences between the image cubes. These differences are due to variations in: target characteristics, solar illumination, atmospheric conditions and errors of the imaging system proper*”. Peleg et al. (2005) characterized and quantified levels of radiometric repeatability in optical sensing data acquired both under controlled laboratory conditions with artificial lighting and from airborne remote sensing missions (sun as light source). We are unaware of any similar research articles, in which temporal radiometric repeatability has been experimentally tested and accompanied by statistical analyses. The fundamental issue is that training data used to develop classification functions should ideally include acquisitions of remote sensing data at more than one time point. Furthermore, performance validation of optical classifications should ideally be based on truly independent data (Nansen et al., 2022).

**Figure 2:**
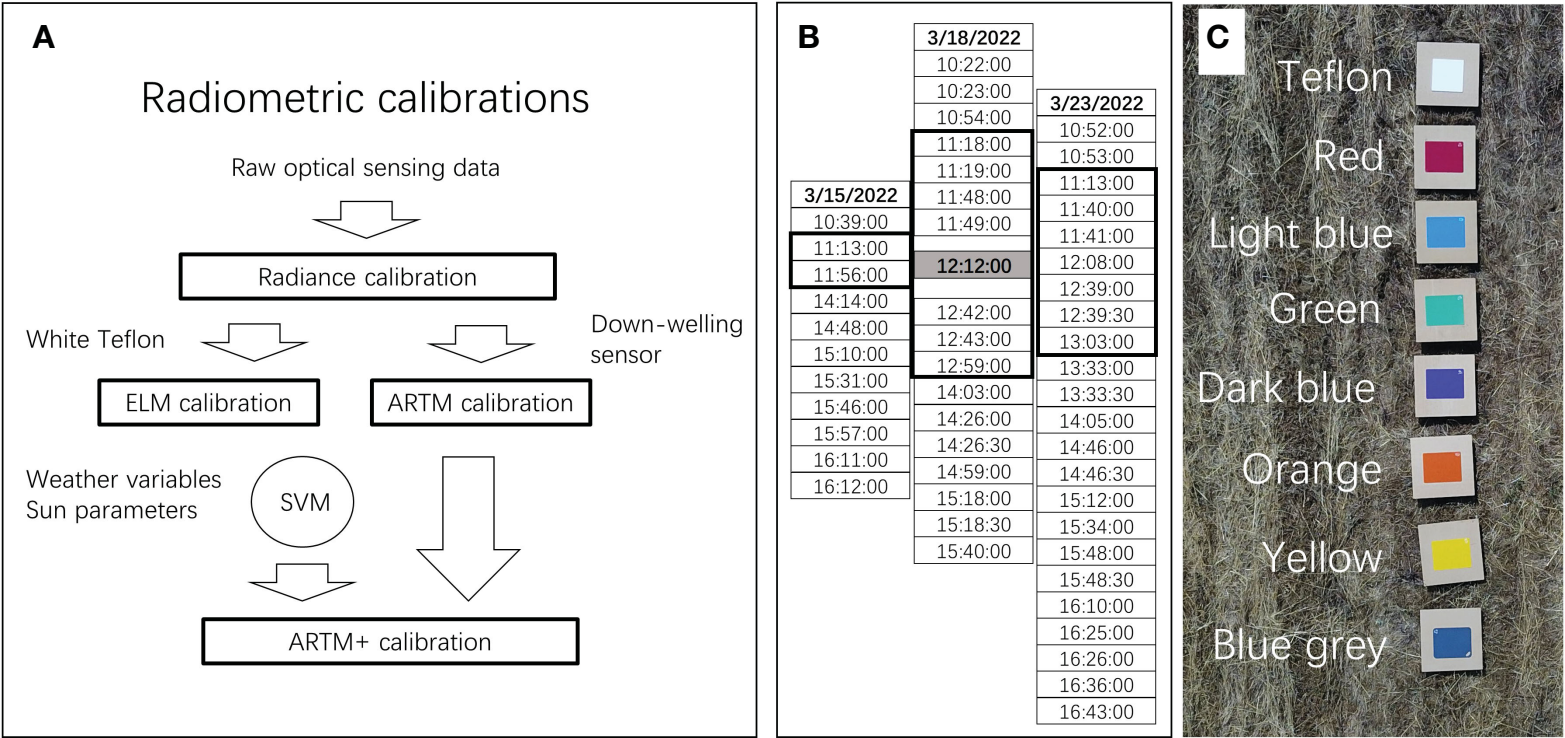
Illustration of data work flow related to radiometric calibration of optical sensing data **(A)**. Time of 52 flight missions on three separate days **(B)**. Optical sensing data acquired during one flight mission (Day 2, 12:12) is highlighted as it was used as to develop classification functions (one for each level of radiometric calibration), which were applied to the remaining 51 optical sensing data sets. Experimental objects (white Teflon and seven colored panels) placed on top of wooden boards and imaged during 52 flight missions **(C)**.

In this study, we acquired airborne hyperspectral optical sensing data from experimental objects (white Teflon and colored panels) during 52 flight missions on three separate days. The following variables were controlled/fixed during flight missions: 1) position of experimental objects (placed always in same positions and sequence), 2) type of experimental objects (exact same objects used in during all flight missions), 3) quality of objects (as they were plastic, we assumed negligible change during course of this study), 4) distance and angle of imaging lens in relation to objects (drone altitude, speed, and linear trajectory were programmed and assumed to be the same for all flight missions), and 5) camera settings and imaging (e.g. exposure time and frame rate) so that hyperspectral images were acquired under constant settings and therefore directly comparable. However, the following variables were not controlled during and among flight missions: sun parameters (altitude, azimuth, and distance from earth), cloud cover, atmospheric composition, and weather conditions. We tested the hypothesis that radiometric calibration, encompassing non-linear dynamics of sun parameters and weather conditions, can significantly improve radiometric repeatability. To address this hypothesis, three separate data analyses were performed. Firstly, we compared radiometric repeatability of average profiles, when imaging data were subjected to different levels of radiometric calibration [radiance calibration (no radiometric calibration), ELM calibration, and ARTM and ARTM+ (**Figure 2A**). Importantly, Analysis 1 focused on the overall repeatability among levels of radiometric calibration. Furthermore, Analysis 1 was used to identify spectral regions with comparatively low/high radiometric repeatability. That is, insight into portions of the radiometric spectrum with comparatively high radiometric repeatability can be used to select spectral bands from such regions and thereby optimize likelihood of high overall performance of classification functions. While Analysis 1 was performed to characterize the overall level of radiometric repeatability, Analysis 2 examined radiometric repeatability from a different perspective, as optical sensing data acquired close to zenith on day 2 (**Figure 2B** at 12:12 pm on March 18) were used to develop a classification function of pixels from the seven color panels. Subsequently, this classification function was applied to the remaining 51 optical sensing data sets acquired earlier and later the same day and also on days before and after. Thus, Analysis 2 represented a scenario, in which a classification model based on optical sensing data from a single flight mission was applied to data from all other flight missions and therefore provided insight into “robustness” (Nansen, 2011) of the classification model. High level of robustness (similar classification results among optical sensing data sets would provide strong indication of high repeatability due to high-performance of radiometric calibration. In Analysis 2, the fundamental assumption was that numbers pixels classified as one of the seven panels should remained similar over time (within and between days of flight missions). Accordingly, variation in number of pixels classified as each panel among flight missions was interpreted using Equation 1 and therefore used as proxy of radiometric repeatability. As Analysis 3, a classification function of pixels from the seven color panels was developed, in which spectral bands with low radiometric repeatability had been omitted. Meaning the level and type of radiometric calibration was identical to that of Analysis 2, but the number of spectral bands included to develop classification functions had been optimized to only include those with high spectral repeatability (derived from Analysis 1).

Based on experimental airborne optical sensing data and three separate analyses, this study confirms what Peleg et al. (2005) eloquently highlighted as a major issue being ignored in most remote sensing studies – that optical sensing data acquired from the same object at multiple time points show considerable variability. Thus, the issue of temporal radiometric repeatability is highlighted, as most published optical sensing studies lack repetition over time of data acquisitions. Results from this study highlight concerns about radiometric repeatability as being relevant, even when optical sensing data are used to classify objects distinguishable by the human eye. We argue that this study is relevant to virtually all studies involving applications of airborne remote sensing.

## Materials and methods

### Drone-based imaging of experimental objects

A total of 52 flight missions were completed (**Table 1** and **Figure 2B**), in which we acquired optical sensing data from white Teflon and seven color panels (red, light blue, green, dark blue, orange, yellow, and blue grey) (**Figure 2C**). We used a push-broom hyperspectral camera (PIKA L, Resonon Inc., Bozeman, MT, USA) with the following specifications: digital output (12 bit), angular field of view of 7 degrees, objective lens had a 17 mm focal length (maximum aperture of F1.4), spectral range of 380-1,015 nm, and spectral resolution of 150 bands (4.2 nm). However, we only analyzed reflectance values in 131 spectral bands from 416 -970 nm due to concerns about low signal to noise ratio in both ends of the spectral range. The hyperspectral camera was mounted on a gimbal (DJI Ronin-M Model R-6, DJI, Shenzen, China) and flown with an octocopter (DJI S1000 octocopter, DJI, Shenzhen, China), which was controlled using a DJI A3 Pro flight controller and a DJI Lightbridge 2 radio controller. To obtain directly comparable remote sensing data, the following variables were controlled/fixed during flight missions: 1) position of experimental objects (placed always in same positions and sequence), 2) type of experimental objects (exact same objects used in during all flight missions), 3) quality of objects (as they were plastic, we assumed negligible change during course of this study), 4) distance and angle of imaging lens in relation to objects (drone altitude = 30 meters, speed = 1.3 meters per second, and linear trajectory were programmed and assumed to be the same for all flight missions), and 5) all camera settings were constant and identical for all flight missions (integration time = 3 milliseconds, frame rate = 130 per second, and gain = 0).

**Table 1:** Weather variables and sun parameters and during flight missions on three separate days.

	3/15/2022	3/18/2022	3/23/2022	Repeatability
**Flight time (first-last)**	10:39-16:12	10:22-15:40	10:52-16:43	
**Number of flights**	11	18	23	
**Wind speed**	1.35 (0.00-3.02)	0.66 (0.00-2.52)	0.88 (0.00-2.01)	74.43
**Temperature °C**	22.07 (21.73-26.35)	18.46 (17.03-20.56)	24.61 26.40-29.17	95.98
**Relative humidity (%)**	55.58 (39.78-43.21)	44.70 (31.40-37.80)	75.30 (54.70-54.40)	94.07
**Barometric pressure**	1.02 (1.02-1.02)	1.02 (1.01-1.02)	1.02 (1.02-1.02)	99.94
**Altitude**	39.31 (32.82-47.42)	44.00 32.54-50.58	43.40 29.46-52.65	95.85
**Azimuth**	201.01 (128.86-235.77)	173.42 (121.87-229.15)	195.05 (130.33-245.43)	94.15
**Distance**	148.79 (148.78-148.79)	148.91 (148.90-148.91)	149.12 (149.11-149.12)	99.98

Average, and minimum and maximum (inside brackets) weather variables: wind speed (m/s), ambient temperature, relative humidity, and barometric pressure (bar). Average, and minimum and maximum (inside brackets) sun parameters, and data for all 52 flight missions were obtained a from website (https://www.suncalc.org/): altitude (deg), azimuth (deg), and distance (million km) from earth. Repeatability was calculated based on Equation 1 and data from all 52 flight missions.

Hyperspectral images were acquired with a spatial resolution of about one pixel per cm^2^. For each combination of flight mission and white Teflon or colored panel, we selected about 225 central pixels (15 × 15 = 225), which were averaged as a single hyperspectral profile. To ensure identical sequence, position, and to avoid dust deposition during flight missions, white Teflon and colored panels were cleaned between flight missions and placed on top of cinder blocks. A down-welling sensor (Flame-S, Ocean Insight, Orlando, FL) was mounted on top of the drone and used to acquire irradiance data (**Figure 1B**), and it was connected to the drone computer with a fiber-optic cable. The following four weather variables were acquired with a ground-based weather station (HOBO U30 Station, Onset, Bourne, MA): wind speed (m/s), ambient temperature (°C), relative humidity (%), and barometric pressure (bar) during all flight missions. The following three sun parameters were obtained from a website (https://www.suncalc.org/): altitude (deg), azimuth (deg), and distance (km) from earth.

### Data analysis

All data processing and classifications were performed in R v3.6.1 (The R Foundation for Statistical Computing, Vienna, Austria). Optical sensing data from the 52 flight missions were subjected to four levels of radiometric calibration [radiance calibration (no radiometric calibration), ELM calibration, and ARTM and ARTM+ calibrations]. These radiometric calibration methods are illustrated in **Figure 2A** and briefly described in the workflow below:

- Raw hyperspectral imaging data from both white Teflon and colored boards and irradiance down-welling sensor data were acquired simultaneously with flying drone during 52 flight missions.
- Using Spectronon Pro software (www.resonon.com), raw hyperspectral imaging data from colored panels were converted into radiance, and this represented the first radiometric calibration level, which is referred to as “radiance calibration”.
- Using irradiance data from on-board down-welling sensor, radiance optical sensing data were converted into relative reflectance. This level of radiometric calibration was performed using the software, Spectronon Pro (www.resonon.com), and it is referred to as “ARTM”.
- Averaged radiance profiles from all combinations of flight mission and colored panel were divided with averaged radiance profiles from corresponding white Teflon. This level of radiometric calibration is referred to as “ELM”.
- Data for seven explanatory variables (four weather variables and three sun parameters) were obtained for the time period of each of the 52 flight missions, and support vector machine (svm) modeling [using the library(e1071) with radial kernel function and no specific hyperparameters (i.e., cost or gamma)] was performed with reflectance data from white Teflon. Separate svm models were performed with reflectance signals in 131 individual spectral bands. These svm model outputs may be considered virtual reference calibration boards, and reflectance data from color panels were divided with svm model outputs. This level of radiometric calibration is referred to as “ARTM+”, as it included calibration based on both irradiance signals from the on-board down-welling sensor and from svm modeling of sun parameters and weather variables.

Three separate analyses were performed based on the abovementioned levels of radiometric calibration of optical sensing data. In Analyses 1 and 3, highly similar results were obtained with all seven color panels. Accordingly, only results from a single color panel (red panel) are presented. Regarding Analysis 2, results from all seven color panels are presented.

Analysis 1: We quantified radiometric repeatability as a function of level of radiometric calibration of average profiles from the 52 flight missions. Equation 1 was used to generate radiometric repeatability estimates for all combinations of radiometric calibration method (four methods) and spectral bands (131 spectral bands). This analysis represents a *post-hoc* (after completion of all flight missions) characterization and assessment of the overall level of radiometric repeatability.

Analysis 2: Optical sensing data acquired at 12:12 pm on March 18 were used to develop classification functions (one for each level of radiometric calibration) of pixels from the seven color panels. These classification functions were applied to the 51 remaining optical sensing data sets acquired earlier and later the same day and also on days before and after. Thus, we take an analytical approach which is similar to what would be deployed by optical sensing practitioners when an existing classification function (with corresponding radiometric calibration) is used and applied to “new” optical data sets. In addition to categories representing each of the seven color panels, we included categories representing: wooden board underneath color panels, and soil/vegetation. Svm modeling [using the library(e1071) with radial kernel function and no specific hyperparameters (i.e., cost or gamma)] was used to generate classification function based on optical sensing data from 12:12 pm on March 18. In Analysis 2, the fundamental assumption was that numbers pixels classified as one of the seven panels should remain similar over time (within and between days of flight missions). Accordingly, variation in number of pixels assigned to each panel among flight missions was interpreted using Equation 1 and therefore used as proxy of radiometric repeatability. We used paired t-test (library(rstatix)) for statistical comparisons of average radiometric repeatability values for the seven color panels among levels of radiometric calibration. This analysis enabled direct comparison and assessment of effect of calibration on radiometric repeatability. Additionally, 2^nd^ order polynomial regression analyses were performed for optical sensing data acquired with each of the four levels of radiometric calibration. In these regression analyses, azimuth (deg) of each flight mission was used as explanatory variable, while number of pixels (only data from green color panel are presented) was used as response variable. A straight regression with slope close to zero would imply low sensitivity of pixel numbers correctly classified to weather variables and sun parameters at time of flight missions. Conversely, a unimodal regression fit would indicate high sensitivity of pixel numbers correctly classified to abiotic conditions at time of flight missions.

Analysis 3: the same analytical approach as described in Analysis 2 was applied to all four radiometric calibration levels, but instead of using all 131 spectral bands (416-970 nm) we only used 115 spectral bands from (416-900 nm), thus excluding spectra bands >900 nm, as these were found to be associated with comparatively low radiometric repeatability during Analysis 1.

## Results

### Analysis 1 - radiometric repeatability of average profiles

**Figure 3:**
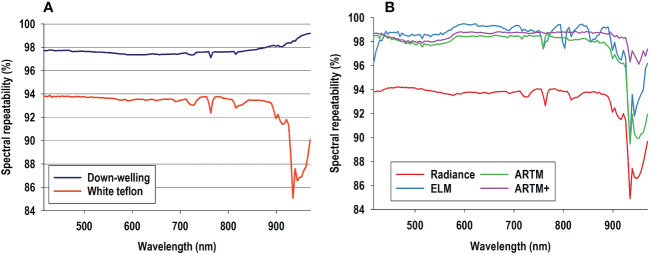
Equation 1 was used to calculate spectral repeatability of irradiance profiles (acquired with drone-mounted down-welling sensor) and of radiance profiles from white Teflon from 52 flight missions **(A)**. Equation 1 was also used to calculate spectral repeatability of profiles from red color panel based on four levels of spectral calibration **(B)**: 1) no spectral calibration (radiance data), 2) spectral calibration based on white calibration boards (empirical line method, ELM calibration), 3) spectral calibration with irradiance data acquired with a drone-mounted down-welling sensor (ARTM), and 4) modeled sun parameters and weather variables in combination with irradiance data from drone-mounted down-welling sensor (ARTM+).

**Table 1** lists minimum, maximum, and average times of the 52 flight missions, and we used Equation 1 to calculate repeatability of decimal flight times. Similar repeatability values were calculated for sun parameters and weather variables, and it is seen that all variables, except wind speed, were associated with repeatability scores between 94-100. In other words, only about 5% of data sets would be expected to fall outside 95% confidence intervals of these variables. Regarding down-welling irradiance data, radiometric repeatability values ranged from 95-98% for spectral bands in the examined radiometric spectrum (**Figure 3A**). In other words, only 2-5% of down-welling sensor data would be expected to fall outside 95% confidence intervals of average values. Radiometric repeatability values of radiance calibration acquired from the white Teflon board were around 94% in spectral bands from 416-900 nm and decreased markedly in spectral bands from about 900-970 nm (**Figure 3A**). It is highly noteworthy that radiometric repeatability values of radiance calibration data acquired from the white Teflon board were consistently lower than those of irradiance data and similar to those of sun parameters and weather variables. This may be interpreted as variables other than sun light intensity (irradiance) markedly influencing radiance data acquired with a flying drone.

**Figure 3B** shows average radiometric repeatability profiles from the red color panel in response to four different levels of radiometric calibration. Only data from the red color panel are shown, but very similar results were observed for other six color panels. It is seen that radiometric repeatability of radiance calibration from the red color (**Figure 3B**) was almost identical to that of white Teflon (**Figure 3A**). As expected, all three calibration methods greatly improved radiometric repeatability compared to non-calibrated radiance data, and they all showed similar radiometric repeatability as irradiance data, except for spectral bands beyond 900 nm. This decrease suggests spectral bands in this portion of the radiometric spectrum may be associated with considerable stochastic noise and therefore adversely affect performance of classification functions. Importantly, ARTM+ calibration yielded considerably higher radiometric repeatability in spectral bands beyond 900 nm than other radiometric calibration methods.

### Analysis 2 - radiometric repeatability of classified color panels

**Figure 4:**
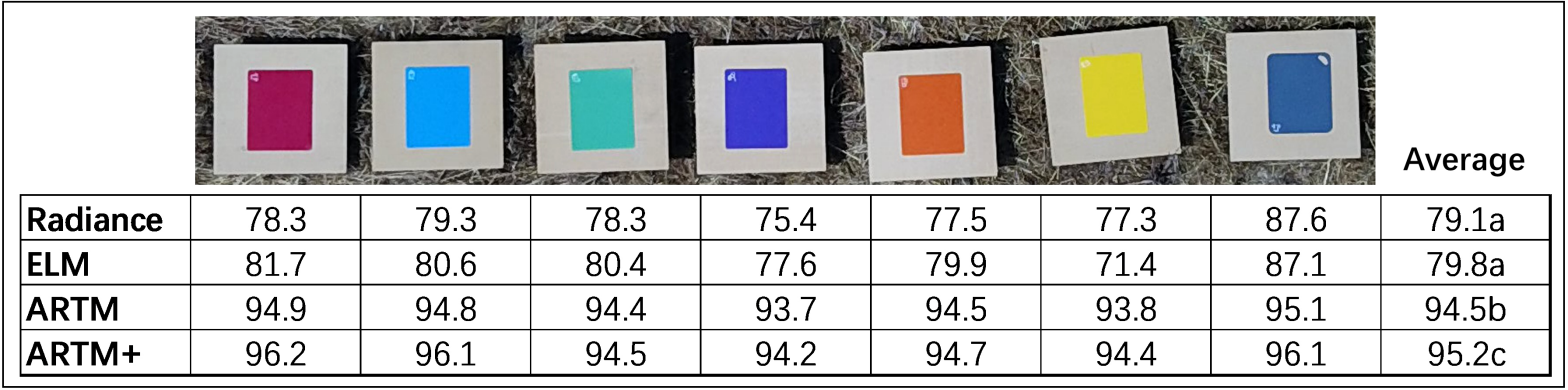
Classification functions derived from optical remote sensing data acquired from colored panels during one flight mission (Day 2, 12:12) were used to develop classification functions for each of the four levels of spectral calibration. Subsequently, these classification functions were applied to hyperspectral remote sensing data acquired during each of the 52 flight missions. Numbers of correctly classified pixels were used as proxy to estimate spectral repeatability for each color panel. Letters indicate statistical difference at the 0.05-level.

We are unaware of any published studies in which one classification function is truly validated based on 51 additional optical sensing data sets. The seven color panels were visibly distinguishable, so, as expected, svm classification functions of data from all four levels of radiometric calibration yielded classification accuracies exceeding 98% (based on 10-fold validation) (classification results not shown). Meaning, irrespectively of level of radiometric calibration, it could be concluded that each classification function (based only on remote sensing data acquired from a single flight mission at 12:12 on March 18), irrespectively of radiometric calibration method, could be used with similar level of performance accuracy to classify remote sensing data from the remaining 51 flight missions. This issue is what was experimentally tested in this study, as most remote sensing studies ignore the importance of acquiring data from the same objects multiple times and applying classification function to new and independent data sets (Peleg et al., 2005; Anderson and Peleg, 2007). Accordingly, we applied classification functions derived from the flight mission at 12:12 on March 18 to optical sensing data acquired during the remaining 51 flight missions (**Figure 4**). Radiance and ELM calibrations showed significantly lower radiometric repeatability than data subjected ARTM calibrations. In addition to low average radiometric repeatability, there was considerable sensitivity to specific colors of panels for both radiance and ELM calibrations. As an example, radiance calibration of the yellow color panel was associated with a radiometric repeatability of 77.3%, while that of the blue grey panel was 87.6%. Similarly for ELM calibration, the yellow color panel was associated with a radiometric repeatability of 71.4%, while that of the blue grey panel was 87.1%. This inconsistency among color panels means that classification accuracies appeared to be highly sensitive to colors of target objects and therefore of optical traits of categories included in classification functions. In contrast, both ARTM calibrations yielded highly consistent average radiometric repeatability values for all seven color panels, and overall radiometric repeatability values around 95% with ARTM+ calibration being significantly higher than ARTM calibration (df = 6, t-stat = 3.742, P = 0.010).

**Figure 5:**
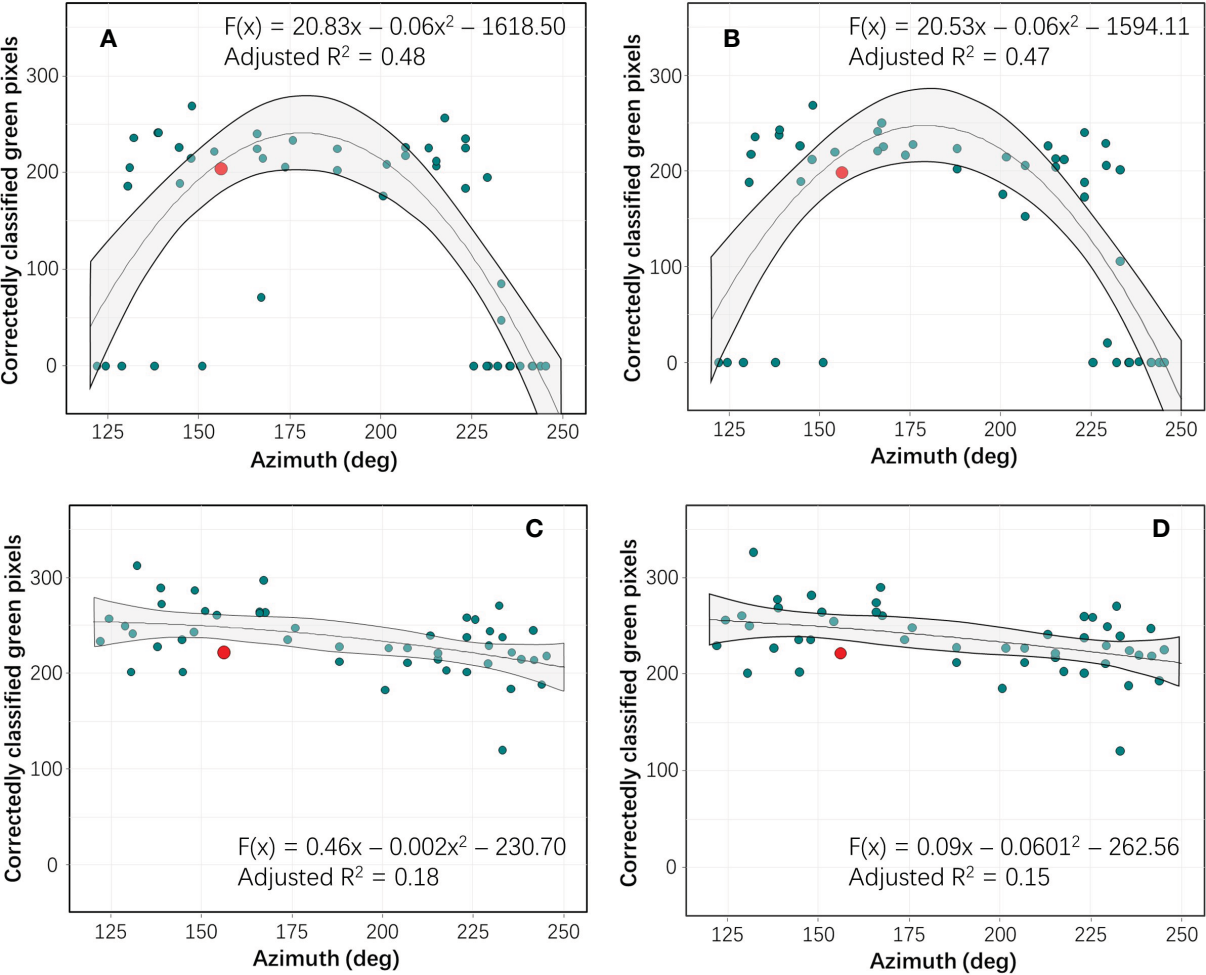
Based on remote sensing data acquired from green color panel (considered representative for all seven color panels), numbers of correctly classified pixels are plotted as a function of azimuth (deg) for each of the four levels of spectral calibration (red dots denote time point of data used to generate classification function): 1) no spectral calibration (radiance data) **(A)**, 2) spectral calibration based on white calibration boards (empirical line method, ELM calibration) **(B)**, 3) spectral calibration with irradiance data acquired with a drone-mounted down-welling sensor (ARTM) **(C)**, and 4) modeled sun parameters and weather variables in combination with irradiance data from drone-mounted down-welling sensor (ARTM+) **(D)**. In each graph, hyperspectral remote sensing data acquired during one flight mission (Day 2, 12:12) are presented as a white dot, as this was used to training data for the classification function applied to data from all 52 flight missions. A second order polynomial regression fit was deployed to each data set, and grey areas denote 95% confidence intervals.

With flight missions performed across wide time spans on each of three days, we examined effects of time difference from model data acquisition (12:12 pm). For each of the 52 flight missions, number of pixels correctly classified as green color panel was determined and used to calculate radiometric repeatability scores (green color panel was representative of all color panels). We observed that, regarding radiance (**Figure 5A**) and ELM calibration (**Figure 5B**), the green color panel was represented by approximately 200 pixels in data sets acquired with azimuth near 150°. However, classification functions failed to identify any pixels correctly as green color panel (green pixels = 0) in many of the data sets acquired with azimuth below 130° or above 235°. Instead, pixels from green color panel (and those from five other color panels: red, light blue, dark blue, orange, and yellow) were misclassified as wood board, ground, and blue grey panel. Consequently, there was as strong unimodal response of green panel pixels to time of day for both radiance and ELM calibration. Conversely, both ARTM calibrations showed low sensitivity to azimuth and therefore to time of day and other sun parameters and environmental variables (**Figures 5C, D**). Moreover both polynomial regression slopes were almost linear and with a negative slope, ARTM = -0.18 and ARTM+ = -0.15. Thus, in direct comparison, ARTM+ calibration showed slightly less sensitivity to sun parameters and environmental variables.

### Analysis 3 – improved radiometric repeatability of ARTM2+ classification

**Figure 6:**
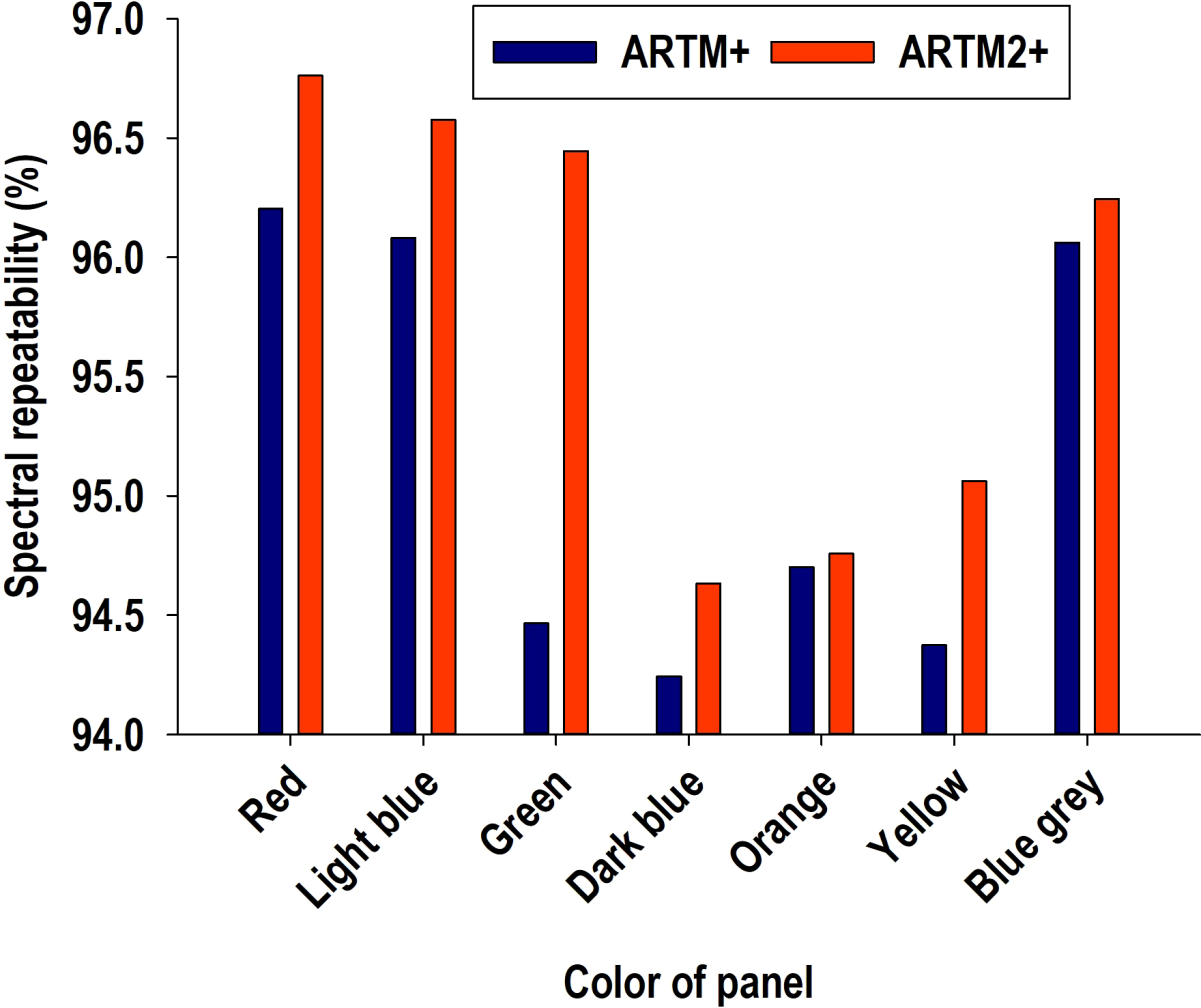
Direct comparison of average spectral repeatability of ARTM+ and ARTM2+ calibrations for each of the seven color panels. ARTM+ calibration is based on data from 131 spectral bands from 406-970 nm, while ARTM2+ calibration is based on data from 115 spectral bands from 406-900 nm.

Curves in **Figure 3B** provided indication of spectral bands beyond 900 nm possibly adversely affecting radiometric repeatability. Consequently, data derived from ARTM+ calibration were re-classified with a subset of 115 spectral bands (excluding 16 spectral bands from 900-970 nm from the classification function), and this radiometric calibration was denoted ARTM2+ (**Figure 6**). Regarding the seven color panels, we observed a small but significant improvement of average radiometric repeatability (df = 6, t-stat = 2.447, P = 0.033).

## Discussion

As a crucial prerequisite to successful and meaningful use of airborne remote sensing data (Anderson and Peleg, 2007) and satellite imagery (Biggar et al., 1994), radiometric calibration methods are needed, so that data acquired multiple times from target objects and/or landscape features are radiometrically repeatable and therefore can be accurately detected and classified. Despite widespread acknowledgement of radiometric calibration as critically important and this topic being the main focus of a large body of research studies, temporal aspects, and therefore temporal radiometric repeatability, have been ignored. Virtually in all experimental research disciplines, replication (both in space and time) is considered a fundamental pillar, and it is required when statistical procedures are deployed. Temporal replication of observations is particularly important when response variables (such as optical signals) are known to be highly influenced by complex and non-linear interactions among abiotic and environmental variables. Accordingly, we tested the hypothesis that radiometric calibration, encompassing non-linear dynamics of sun parameters and weather conditions, can significantly improve temporal radiometric repeatability. For direct comparison of remote sensing data acquired over time, all camera and imaging settings (exposure time, frame rate, lens aperture, and drone speed) were kept constant.

The following are considered main findings of this study: 1) Radiance data acquired from white Teflon showed consistently lower radiometric repeatability than simultaneously acquired irradiance data. Accordingly, irradiance data may only partially account for optical noise in airborne remote sensing data. 2) Spectral bands beyond 900 nm were found to be associated with disproportionally lower radiometric repeatability than signal values in spectral bands from 416-900 nm. This important result implies that inclusion of spectral bands from 900-970 nm may adversely affect accuracy and robustness of classification functions. Reviews of spectral indices show that spectral bands from 900-970 nm are frequently used (Bolin et al., 1989; Thenkabail et al., 2000; Thorp and Tian, 2004; Zhu et al., 2008; Prabhakar et al., 2012; Luo et al., 2013). 3) As expected, radiance data were highly sensitive to time of flight missions (which is directly linked to sun parameters and weather variables). However, we also demonstrated that ELM calibration showed similar time sensitivity. This result raises some concern, as it is probably the most commonly used method of radiometric calibration in airborne remote sensing studies (Aasen et al., 2018; Poncet et al., 2019). 4). We examined two methods of ARTM calibration, in which irradiance data were acquired with a drone mounted down-welling sensor by itself (ARTM), and in which sun parameters and weather variables were modeled and integrated into radiometric calibration (ARTM+). Both ARTM calibration methods outperformed ELM calibration, especially ARTM+. Importantly, ARTM+ calibration markedly attenuated loss of radiometric repeatability in spectral bands beyond 900 nm, and therefore improved possible contributions of these spectral bands to classification functions. Nevertheless, we demonstrated experimentally that exclusion of spectral bands beyond 900 nm caused a significant increase in radiometric repeatability. However, it is important to emphasize that the latter result is highly specific to objects being classified (classes in classification function) and may therefore not be broadly applicable and relevant. 5) A unique element of this study was that the radiometric repeatability measurement (Equation 1) was used to directly compare performances of different radiometric calibration methods. Several studies have proposed other but somewhat similar ways to calculate radiometric repeatability (Kollenkark et al., 1982; Peleg et al., 2005; Anderson and Peleg, 2007; Poncet et al., 2019). Most of these radiometric repeatability calculations are based on quantification of “true reflectance”, which is typically determined by means of reflectance under highly controlled laboratory conditions (inside optical sphere or hemisphere and with controlled lighting). Once true reflectance is known for each spectral band, band-specific RMSE-values (root-mean-square error) can be calculated. It may not always be practically feasible to obtain “true reflectance” values for all combinations of color panels and spectral bands, so a different estimate of radiometric repeatability was used in this study, and several features may be highlighted:

- Based on assumption of optical sensing data following a normal distribution, and use of confidence interval provides an intuitive interpretation of expected frequency distribution of optical signal values.
- Is easy to calculate and can be used to calculate repeatability of all types of optical sensing data
- Due to standardization (division with average signal), it is possible to directly compare radiometric repeatability values among spectral bands with different optical signal strength
- It is scaled so that maximum = 100, which is a convenient presentation of radiometric repeatability.

The proposed radiometric repeatability measure was applied to exact same optical sensing data cubes after being processed according to four different radiometric calibrations. Moreover, slight stochastic variations in flight path, and drone speed and altitude may partially contribute to error in calculations of radiometric repeatability, but these sources of error affected the raw optical sensing data, so radiometric repeatability measures were directly comparable.

### ELM calibration

Use of ELM calibration is probably the most commonly used method of radiometric calibration of airborne remote sensing data (Poncet et al., 2019), and it has been thoroughly reviewed (Smith and Milton, 1999; Baugh and Groeneveld, 2008; Aasen et al., 2018). Several studies have provided important insight into practical use and repeatability of ELM calibration (Che and Price, 1992; Smith and Milton, 1999; Karpouzli and Malthus, 2003; Wang and Myint, 2015; Aasen et al., 2018; Iqbal et al., 2018; Mafanya et al., 2018; Agapiou, 2020; Zarzar et al., 2020). Experimental data from this study showed its considerable sensitivity to time of day (sun parameters and weather variables). Additionally, ELM calibration has the major practical disadvantage that reference boards need to be placed within acquired imaging scenes and sufficiently frequent to account for temporal variations in atmospheric conditions and sun parameters. Both placement and retrieval of calibration boards may be time and labor consuming when large areas are being subjected to airborne remote sensing. Furthermore, calibration boards need to be placed in ways that minimize radiometric noise due to project angle issues and/or shadows being cast by adjacent objects. Calibration boards must be kept clean, stored properly, and undamaged, and they are only of limited practical feasibility in remote sensing studies of tall and dense vegetation or objects. High-quality calibration boards are often costly, which may pose economic constraints to commercial operations and/or large-scale research studies. Additionally, Assmann et al. (2018) suggested that continuous use of commercially available calibration boards (based on supplier and material) under field conditions may lead to change in their optical characteristics over time and therefore compromise their consistency. We are therefore encouraging fellow researchers and commercial practitioners acquiring and classifying airborne remote sensing data to consider integration of ARTM calibration methods. Accurate ARTM calibration may be hampered when based on theoretical calculation of incident irradiance (Smith and Milton, 1999), but use of drone systems with on-board down-welling sensor mitigates this challenge.

### Radiometric repeatability of living objects

In this study, color panels were visibly distinguishable, so virtually any classification function would be able to provide high classification accuracy of data from any of the 52 individual data sets and irrespectively of radiometric calibration method. However in many optical sensing studies, classes are less distinct, such as, studies involving optical sensing of living plants. In addition to classes being less distinct, physiological plant dynamics may influence radiometric repeatability of optical sensing data. This is in itself a considerable challenge, which is further exacerbated when airborne remote sensing data are used to classify living objects, such as plants. Moreover, it has been shown in a number of plants, including tomato (Meyer et al., 1989), petunia (Stayton et al., 1989), tobacco (Paulsen and Bogorad, 1990), and wheat (Nagy et al., 1987; Busheva et al., 1991) that concentration of leaf pigments follow both diurnal and circadian rhythms. Thus, light absorption should not be expected to be constant over time, is influenced by plant ontology and phenology, and may vary among leaves on the same plant as a function of leaf age, cardinal direction, and canopy structure. Furthermore, it has been demonstrated that in plant chloroplasts move and are near outer leaf surface during the day, which results in high absorbance of radiometric energy, while they are mainly located along leaf sides at night (Britz and Briggs, 1976). Actual impact of such physiological dynamics on leaf reflectance have, to the best of our knowledge not been thoroughly examined, but they provide insight into possible factors adversely affecting radiometric repeatability of optical sensing data from plants.

### Studies of radiometric repeatability

Some studies examining performance of different radiometric calibration methods are based on remote sensing data acquired during a single flight mission (Smith and Milton, 1999; Iqbal et al., 2018; Shin et al., 2020). Some studies have examined radiometric repeatability as a function of specific variables. As an example, Daughtry et al. (1982) compared optical sensing data acquired at 10 altitudes ranging from 0.2 to 10 m above maize and soybean fields with varying degree of soil cover. In both red (600-700 nm) and infrared (800-1100 nm) portions of the radiometric spectrum, the authors found a positive association between altitude and radiometric repeatability. Flying at higher altitude also means that slight changes (i.e. due to wind) in vertical position of flying drones has proportionally less influence on spatial resolution of optical data then flying at lower altitude. However, flying at higher altitudes means reduction in spatial resolution and therefore increase in mixed pixels, and that may adversely affect radiometric repeatability. Zarzar et al. (2020) acquired drone-based remote sensing data from grey calibration boards at nine altitudes ranging from 4 – 244 m, and they developed an ELM calibration framework to maximize radiometric repeatability based on atmospheric correction. The study by Zarzar et al. (2020) was based on remote sensing data acquired during multiple flight missions over the course of a full year, and goodness of fit values for three spectral bands were: R^2^-green = 0.77, R^2^-red = 0.79, and R^2^-nir = 0.77). Based on number of observations, the fact that the study was conducted over water, and level of temporal replication, obtained goodness of fit values suggest strong performance of the proposed ELM calibration. Using a Landsat radiometer, Kollenkark et al. (1982) examined soybean canopy reflectance in 15-min intervals on three separate days. The authors found that, in the red portion of the radiometric spectrum (600-700 nm), diurnal crop reflectance varied as much as 140%. Furthermore, they showed a clear unimodal diurnal crop reflectance to time difference from azimuth, which was also found in the current study regarding radiance and ELM calibration (**Figures 5A, B**). A correction model was proposed based on row width, row direction, and solar azimuth and zenith angle. Kollenkark et al. (1982) provided a very comprehensive review of models to correct for solar angle in remote sensing studies of vegetation, and most of the models hinge on shadow effects.

Similar to the current study, Iqbal et al. (2018) acquired remote sensing data from color panels, but no validation was included, nor did they quantify or assess relative effects of weather variables and/or sun parameters. Assmann et al. (2018) provided a comprehensive description of sources of error and also specific recommendations to optimize acquisition of airborne remote sensing data with a specific multispectral camera. Interestingly, Assmann et al. (2018) mentioned and briefly described a study with two arctic field sites, and from which airborne remote sensing data were acquired four times on a single day. Furthermore, they examined and quantified relative contributions of five different sources of radiometric error and concluded that cumulative error was approximately 10%–13% of peak vegetational reflectance. Thus, although based on a considerably smaller data set, a different sensor, and acquired under very different environmental conditions, radiometric error estimates by Assmann et al. (2018) are similar in magnitude as observed in this study. Anderson and Peleg (2007) provided a comprehensive description of variables contributing to loss of radiometric repeatability, even between remote sensing data acquired a few minutes apart. However, no statistical analyses were included to quantify effects of time. The challenge associated with low radiometric repeatability has also been demonstrated based on consistency over time of optical data acquired from carefully selected target objects (spectrally homogeneous, Lambertian and horizontal, and at least 12×12 m (Karpouzli and Malthus, 2003). Using ground truthing data from these target object over a period of nine days, the authors found that optical data from calibration targets ranged from 6% to 21% (based on coefficient of variation). In other words, Karpouzli and Malthus (2003) obtained radiometric repeatability levels, which are directly comparable with radiometric repeatability values presented in this study. Additionally, they demonstrated the worrisome issue that radiometric repeatability may be sensitive to and vary among specific target objects. The current study also highlighted an issue with regards to ELM calibration. Poncet et al. (2019) compared five radiometric calibrations, in which three involved a down-welling irradiance sensor, and multispectral airborne remote sensing data were acquired from grey panels with reflectance ranging from 5%-90%. Additionally, this study included flight missions on seven separate days, and they deployed a ground-based weather station to acquire weather data during flight missions. Thus, in several important ways the experimental design was similar to the current study. Poncet et al. (2019) used their data to compare performance of radiometric calibrations and to assess spectral repeatability in each of the four spectral bands (green, red, red-edge, and NIR). Although weather data were acquired, Poncet et al. (2019) did not integrate weather data into any of their five radiometric calibration methods, nor did the authors perform statistical comparison of radiometric calibrations. Very interestingly, Poncet et al. (2019) calculated, for all combinations of multispectral band and radiometric calibration method, percentages of pixels from validation targets which were within validation ranges. They found that for green and red spectral bands,<50% of pixels were within validation ranges, while for red-edge and NIR spectral bands most pixels were within validation ranges. The authors also concluded that “*the distribution of radiometric error changed from flight to flight, and the magnitude of the differences observed between flights varied between multispectral bands and radiometric calibration methods*.”.

### Atmospheric variables and radiometric repeatability

**Figure 3A** provided strong support for the claim that variables other than solar irradiance are likely contributing markedly to loss of radiometric repeatability. **Table 1** shows considerable variation in relative humidity at ground level. We did not have access to vertical atmospheric profile data of gas concentrations, but it is likely variation in atmospheric composition contributed to loss of radiometric repeatability. Importantly, atmospheric gasses, such as 0_4_, 0_2_, H_2_O, NO_2_, and 0_3_, are known to both vary considerably within short amounts of time (Fowler et al., 2009) and indirectly influence optical sensing signals (Solomon et al., 1998). Importantly, access to vertical atmospheric profile data of gas concentrations may be considered a major practical, logistical, and financial obstacle and therefore highly challenging to incorporate into most types of atmospheric correction. Solomon et al. (1998) showed that atmospheric concentration of these gasses is linked directly to absorption peaks in wavelengths from 400-450 nm and 610-680 nm. However, we did not observe noticeable absorption peaks in those spectral regions. Instead, an absorption peak at 762 nm was noticeable in both solar irradiance data and in radiance data from white Teflon. Importantly, absorption near 760 nm is associated with molecular oxygen in the terrestrial atmosphere (Rascher et al., 2009). Furthermore, ELM and ARTM calibrations retained this absorption peak at 762 nm, but it was eliminated by ARTM+ calibration (**Figure 3B**). The fact that ARTM+ calibration produced a considerably smoother profile than any of the other radiometric calibrations supports the claim that weather variables and atmospheric gas composition are important explanatory variables in efforts to maximize radiometric repeatability.

## Final perspectives

It is indisputable that integration of advanced airborne remote sensing, such as hyperspectral optical sensing, into monitoring and management of crops in agriculture, environmental conservation, and many commercial industry sectors, holds considerable potential (Anderson and Gaston, 2013; Aasen et al., 2018; Assmann et al., 2018; Zarzar et al., 2020). However, widespread adoption of remote sensing technologies is constrained by a critically important challenge – non-linear dynamics of weather, solar light intensity, atmospheric composition, scattering, and how effects of these variables are integrated into radiometric calibrations. As we have highlighted and reviewed, there are reasons to be concerned about use of ELM calibration. Moreover, we have identified both logistical concerns as well as performance concerns associated with ELM calibration. As an alternative to ELM calibration, ARTM calibration with an on-board down-welling sensor provided significantly higher radiometric repeatability and is not accompanied by the same logistical concerns as ELM calibration. ARTM calibration provided particularly high radiometric repeatability when down-welling sensor data were integrated with sun parameters and weather variables. In the present study, we demonstrated that even though objects were visibly distinct (color panels) and therefore very easy to classify, replication of image acquisitions over time resulted in loss of radiometric repeatability with all examined methods of radiometric calibration. Moreover, a minimum of 5% radiometric error (radiometric repeatability<95%) should be expected when airborne remote sensing data are acquired. And as described above, other studies based on different remote sensing systems corroborate this result, or they suggested lower levels of radiometric repeatability. A direct implication is that objects being classified should be in classes that are at least 5% different in terms of average spectral traits for classification functions to perform with high degree of accuracy and consistency.

## Data availability statement

The raw data supporting the conclusions of this article will be made available by the authors, without undue reservation.

## Author contributions

CN conceptualized experiments. AM performed drone missions and corrected hyperspectral image cubes. HL programmed data processing. CN analyzed data. CN and HL wrote manuscript drafts. All authors contributed to the article and approved the submitted version.

## References

[B1] Aasen H. Honkavaara E. Lucieer A. Zarco-Tejada P. (2018). Quantitative remote sensing at ultra-high resolution with uav spectroscopy: A review of sensor technology, measurement procedures, and data correction workflows. Remote Sens. 10, 1091. doi: 10.3390/rs10071091

[B2] Agapiou A. (2020). Vegetation extraction using visible-bands from openly licensed unmanned aerial vehicle imagery. Drones 4, 27. doi: 10.3390/drones4020027

[B3] Anderson K. Gaston K. J. (2013). Lightweight unmanned aerial vehicles will revolutionize spatial ecology. Front. Ecol. Environ. 11, 138–146. doi: 10.1890/120150

[B4] Anderson G. L. Peleg K. (2007). Quantification and reduction of erroneous differences between images in remote sensing. Environ. Ecol. Stat 14, 113–127. doi: 10.1007/s10651-007-0013-4

[B5] Assmann J. J. Kerby J. T. Cunliffe A. M. Myers-Smith I. H. (2018). Vegetation monitoring using multispectral sensors–best practices and lessons learned from high latitudes. J. Unmanned Vehicle Syst. 7, 54–75. doi: 10.1139/juvs-2018-0018

[B6] Baghzouz M. Devitt D. A. Morris R. L. (2006). Evaluating temporal variability in the spectral reflectance response of annual ryegrass to changes in nitrogen applications and leaching fractions. Int. J. Remote Sens. 27, 4137–4157. doi: 10.1080/01431160600851843

[B7] Baugh W. M. Groeneveld D. P. (2008). Empirical proof of the empirical line. Int. J. Remote Sens. 29, 665–672. doi: 10.1080/01431160701352162

[B8] Biggar S. F. Slater P. N. Gellman D. I. (1994). Uncertainties in the in-flight calibration of sensors with reference to measured ground sites in the 0.4-1.1 μm range. Remote Sens. Environ. 48, 245–252. doi: 10.1016/0034-4257(94)90145-7

[B9] Bolin F. P. Preuss L. E. Taylor R. C. Ference R. J. (1989). Refractive index of some mammalian tissues using a fiber optic cladding method. Appl. Optics 28, 2297–2303. doi: 10.1364/AO.28.002297 20555515

[B10] Britz S. J. Briggs W. R. (1976). Circadian rhythms of chloroplast orientation and photosynthetic capacity in ulva. Plant Physiol. 58, 22–27. doi: 10.1104/pp.58.1.22 16659613 PMC542172

[B11] Burkart A. Cogliati S. Schickling A. Rascher U. (2013). A novel UAV-based ultra-light weight spectrometer for field spectroscopy. IEEE sensors J. 14, 62–67. doi: 10.1109/JSEN.2013.2279720

[B12] Busheva M. Garab G. Liker E. Tóth Z. Szèll M. Nagy F. (1991). Diurnal fluctuations in the content and functional properties of the light harvesting chlorophyll a/b complex in thylakoid membranes : correlation with the diurnal rhythm of the mRNA level. Plant Physiol. 95, 997–1003. doi: 10.1104/pp.95.4.997 16668134 PMC1077643

[B13] Carter G. A. Knapp A. K. (2001). Leaf optical properties in higher plants: Linking spectral characteristics to stress and chlorophyll concentration. Am. J. Bot. 88, 677–684. doi: 10.2307/2657068 11302854

[B14] Che N. Price J. C. (1992). Survey of radiometric calibration results and methods for visible and near infrared channels of NOAA-7, -9, and -11 AVHRRs. Remote Sens. Environ. 41, 19–27. doi: 10.1016/0034-4257(92)90057-Q

[B15] Daughtry C. S. T. Vanderbilt V. C. Pollara V. J. (1982). Variability of reflectance measurements with sensor altitude and canopy type. Agron. J. 74, 744–751. doi: 10.2134/agronj1982.00021962007400040034x

[B16] Del Pozo S. Rodríguez-Gonzálvez P. Hernández-López D. Felipe-García B. (2014). Vicarious radiometric calibration of a multispectral camera on board an unmanned aerial system. Remote Sens. 6, 1918–1937. doi: 10.3390/rs6031918

[B17] Fowler D. Pilegaard K. Sutton M. A. Ambus P. Raivonen M. Duyzer J.. (2009). Atmospheric composition change: Ecosystems–atmosphere interactions. Atmospheric Environ. 43, 5193–5267. doi: 10.1016/j.atmosenv.2009.07.068

[B18] Hruska R. Mitchell J. Anderson M. Glenn N. F. (2012). Radiometric and geometric analysis of hyperspectral imagery acquired from an unmanned aerial vehicle. Remote Sens. 4, 2736–2752. doi: 10.3390/rs4092736

[B19] Iqbal F. Lucieer A. Barry K. (2018). Simplified radiometric calibration for UAS-mounted multispectral sensor. Eur. J. Remote Sens. 51, 301–313. doi: 10.1080/22797254.2018.1432293

[B20] Karpouzli E. Malthus T. (2003). The empirical line method for the atmospheric correction of IKONOS imagery. Int. J. Remote Sens. 24, 1143–1150. doi: 10.1080/0143116021000026779

[B21] Kedzierski M. Wierzbicki D. Sekrecka A. Fryskowska A. Walczykowski P. Siewert J. (2019). Influence of lower atmosphere on the radiometric quality of unmanned aerial vehicle imagery. Remote Sens. 11, 1214. doi: 10.3390/rs11101214

[B22] King D. L. Kratochvil J. A. Boyson W. E. (1997). “Measuring solar spectral and angle-of-incidence effects on photovoltaic modules and solar irradiance sensors,” in Conference Record of the Twenty Sixth IEEE Photovoltaic Specialists Conference: IEEE. 1113–1116.

[B23] Kollenkark J. C. Vanderbilt V. C. Daughtry C. S. T. Bauer M. E. (1982). Influence of solar illumination angle on soybean canopy reflectance. Appl. Optics 21, 1179–1184. doi: 10.1364/AO.21.001179 20389828

[B24] Luo J. Huang W. Yuan L. Zhao C. Du S. Zhang J.. (2013). Evaluation of spectral indices and continuous wavelet analysis to quantify aphid infestation in wheat. Precis. Agric. 14, 151–161. doi: 10.1007/s11119-012-9283-4

[B25] Mafanya M. Tsele P. Botai J. O. Manyama P. Chirima G. J. Monate T. (2018). Radiometric calibration framework for ultra-high-resolution UAV-derived orthomosaics for large-scale mapping of invasive alien plants in semi-arid woodlands: *Harrisia pomanensis* as a case study. Int. J. Remote Sens. 39, 5119–5140. doi: 10.1080/01431161.2018.1490503

[B26] Mamaghani B. Salvaggio C. (2019). Multispectral sensor calibration and characterization for sUAS remote sensing. Sensors 19, 4453. doi: 10.3390/s19204453 31615104 PMC6832506

[B27] Meyer H. Thienel U. Piechulla B. (1989). Molecular characterisation of the diurnal/circadian expression of the chlorophyll a/b-binding proteins in leaves of tomato and other dicotyledonous and monocotyledonous plant species. Planta 180, 5–15. doi: 10.1007/BF02411404 24201838

[B28] Nagy F. Kay S. A. Chua N.-H. (1987). “The analysis of gene expression in transgenic plants,” in Plant gene research manual. Eds. Gelvin S. B. Schilperoort R. A. (Dordrecht, The Netherlands: Kluwer Academic Press), 1–29.

[B29] Nansen C. (2011). Robustness of analyses of imaging data. Optics Express 19, 15173–15180. doi: 10.1364/OE.19.015173 21934879

[B30] Nansen C. Elliott N. (2016). Remote sensing and reflectance profiling in entomology. Annu. Rev. Entomology 61, 139–158. doi: 10.1146/annurev-ento-010715-023834 26982438

[B31] Nansen C. Imtiaz M. S. Mesgaran M. B. Lee H. (2022). Experimental data manipulations to assess performance of hyperspectral classification models of crop seeds and other objects. Plant Methods 18, 74. doi: 10.1186/s13007-022-00912-z 35658997 PMC9164469

[B32] Nansen C. Stewart A. N. Gutierrez T. Wintermantel W. M. Mcroberts N. Gilbertson R. L. (2019). Proximal remote sensing to differentiate nonviruliferous and viruliferous insect vectors – proof of concept and importance of input data robustness. Plant Pathol. 68, 746–754. doi: 10.1111/ppa.12984

[B33] Nevalainen O. Honkavaara E. Tuominen S. Viljanen N. Hakala T. Yu X.. (2017). Individual tree detection and classification with UAV-based photogrammetric point clouds and hyperspectral imaging. Remote Sens. 9, 185. doi: 10.3390/rs9030185

[B34] Paulsen H. Bogorad L. (1990). Diurnal and circadian rhythms in the accumulation and synthesis of mRNA for the light-harvesting chlorophyll a/b binding protein. Plant Physiol. 88, 1104–1109. doi: 10.1104/pp.88.4.1104 PMC1055723 16666429

[B35] Peleg K. Anderson G. L. Yang C. (2005). Repeatability of hyperspectral imaging systems - quantification and improvement. Int. J. Remote Sens. 26, 115–139. doi: 10.1080/01431160412331291288

[B36] Poncet A. M. Knappenberger T. Brodbeck C. Fogle M. Shaw J. N. Ortiz B. V. (2019). Multispectral UAS data accuracy for different radiometric calibration methods. Remote Sens. 11, 1917. doi: 10.3390/rs11161917

[B37] Prabhakar M. Prasad Y. G. Rao M. N. (2012). “Remote sensing of biotic stress in crop plants and its applications for pest management,” in Crop stress and its management: Perspectives and strategies. Eds. Venkateswarlu B. Shanker A. K. Shanker C. Maheswari M. (New York, USA: Springer), 517–549.

[B38] Rascher U. Agati G. Alonso L. Cecchi G. Champagne S. Colombo R.. (2009). CEFLES2: the remote sensing component to quantify photosynthetic efficiency from the leaf to the region by measuring sun-induced fluorescence in the oxygen absorption bands. Biogeosciences 6, 1181–1198. doi: 10.5194/bg-6-1181-2009

[B39] Schott J. (2007). Remote sensing: The image chain approach (New York, NY, USA: Oxford University Press).

[B40] Shin J.-I. Cho Y.-M. Lim P.-C. Lee H.-M. Ahn H.-Y. Park C.-W.. (2020). Relative radiometric calibration using tie points and optimal path selection for UAV images. Remote Sens. 12, 1726. doi: 10.3390/rs12111726

[B41] Smith G. M. Milton E. J. (1999). The use of the empirical line method to calibrate remotely sensed data to reflectance. Int. J. Remote Sens. 20, 2653–2662. doi: 10.1080/014311699211994

[B42] Solomon S. Portmann R. Sanders R. Daniel J. (1998). Absorption of solar radiation by water vapor, oxygen, and related collision pairs in the earth's atmosphere. J. Geophysical Research: Atmospheres 103, 3847–3858. doi: 10.1029/97JD03285

[B43] Stayton M. Brosio P. Dunsnuir P. (1989). Photosynthetic genes of petunia (Mitchell) are differentially expressed during the diurnal cycle. Plant Physiol. 89, 776–782. doi: 10.1104/pp.89.3.776 16666620 PMC1055921

[B44] Thenkabail P. S. Smith R. B. Pauw E. D. (2000). Hyperspectral vegetation indices and their relationships with agricultural crop characteristics. Remote Sens. Environ. 71, 158–182. doi: 10.1016/S0034-4257(99)00067-X

[B45] Thorp K. R. Tian L. F. (2004). A review on remote sensing of weeds in agriculture. Precis. Agric. 5, 477–508. doi: 10.1007/s11119-004-5321-1

[B46] Vilaseca M. Schael B. Delpueyo X. Chorro E. Perales E. Hirvonen T.. (2014). Repeatability, reproducibility, and accuracy of a novel pushbroom hyperspectral system. Color Res. Appl. 39, 549–558. doi: 10.1002/col.21851

[B47] Wang C. Myint S. W. (2015). A simplified empirical line method of radiometric calibration for small unmanned aircraft systems-based remote sensing. IEEE J. Selected Topics Appl. Earth Observations Remote Sens. 8, 1876–1885. doi: 10.1109/JSTARS.2015.2422716

[B48] Zarco-Tejada P. J. González-Dugo V. Berni J. (2012). Fluorescence, temperature and narrow-band indices acquired from a UAV platform for water stress detection using a micro-hyperspectral imager and a thermal camera. Remote Sens. Environ. 117, 322–337. doi: 10.1016/j.rse.2011.10.007

[B49] Zarzar C. M. Dash P. Dyer J. L. Moorhead R. Hathcock L. (2020). Development of a simplified radiometric calibration framework for water-based and rapid deployment unmanned aerial system (UAS) operations. Drones 4, 17. doi: 10.3390/drones4020017

[B50] Zhang C. Kovacs J. M. (2012). The application of small unmanned aerial systems for precision agriculture : a review. Precis. Agric. 13, 693–712. doi: 10.1007/s11119-012-9274-5

[B51] Zhu Y. Yao X. Tian Y. Liu X. Cao W. (2008). Analysis of common canopy vegetation indices for indicating leaf nitrogen accumulations in wheat and rice. Int. J. Appl. Earth Observation Geoinformation 10, 1–10. doi: 10.1016/j.jag.2007.02.006

